# Overview of Piezoelectric Biosensors, Immunosensors and DNA Sensors and Their Applications

**DOI:** 10.3390/ma11030448

**Published:** 2018-03-19

**Authors:** Miroslav Pohanka

**Affiliations:** Faculty of Military Health Sciences, University of Defense, Trebesska 1575, Hradec Kralove CZ-50001, Czech Republic; miroslav.pohanka@gmail.com

**Keywords:** acoustic sensor, affinity, anisotropy, biosensor, immunosensor, label free, oscillation, piezoelectric, quartz crystal microbalance, QCM

## Abstract

Piezoelectric biosensors are a group of analytical devices working on a principle of affinity interaction recording. A piezoelectric platform or piezoelectric crystal is a sensor part working on the principle of oscillations change due to a mass bound on the piezoelectric crystal surface. In this review, biosensors having their surface modified with an antibody or antigen, with a molecularly imprinted polymer, with genetic information like single stranded DNA, and biosensors with bound receptors of organic of biochemical origin, are presented and discussed. The mentioned recognition parts are frequently combined with use of nanoparticles and applications in this way are also introduced. An overview of the current literature is given and the methods presented are commented upon.

## 1. Introduction

Piezoelectricity or piezoelectric effect is a physical phenomenon which refers to an ability of a material to produce voltage when mechanically stressed. The effect works in the oppose situation as well. Voltage given to surface of a piezoelectric material causes mechanical stress or oscillation when the voltage is alternating. Anisotropic crystals, that is, crystals without center of symmetry, are typical materials exerting piezoelectricity. Aluminum phosphate (berlinite), aluminum nitride, zinc oxide, crystalized topaz (Al_2_SiO_4_(F, OH)_2_), barium and lead titanate, gallium orthophosphate, quartz (SiO_2_), tartrate tetrahydrate (Rochelle salt), polyvinylidene fluoride, polylactic acids can be mentioned as typical piezoelectric materials [1,2,3,4,5,6,7,8,9,10,11].

Considering an analytical chemistry point of view, the piezoelectricity is well suitable for physical sensors and biosensors construction. The principle of such assay can be introduced by following a simplifying explanation. The biosensor or sensor is excited by alternating voltage given on the surface by two electrodes. The alternating voltage causes mechanical oscillations of crystal and frequency of oscillations is measured as the crystal is put into oscillation circuit [12]. Analyte or any other mass bound on surface of crystal or more precisely on surface of electrodes located on the crystal results in change of oscillation frequency [13]. The change (decay) in frequency is proportional to mass bound the crystal as described by Sauerbreay and coworkers [14,15] but change in medium viscosity can also influence the oscillations as defined by Kanazawa and coworkers [16,17]. 

The piezoelectric platform appears to be ideal for the construction of biosensors. It can simply record affinity interactions without the necessity to apply any specific reagents. On the other hand, sensitivity in micrograms necessary to make measurable change in oscillations and some specific aspects like fragility should be taken into consideration. The number of piezoelectric platforms is quite wide and many papers on the issue have been written. As the number of adaptations is high, the number of used biorecognition parts and designs of the biosensors is high as well. Particular types of biorecognition parts of piezoelectric biosensors are explained to cover the recent development in this field where reviews are quite lacked. In this review, survey of actual applications of piezoelectric biosensors is provided and relevant facts regarding to their use are also mentioned. 

## 2. Piezoelectric Immunosensors 

**Figure 1:**
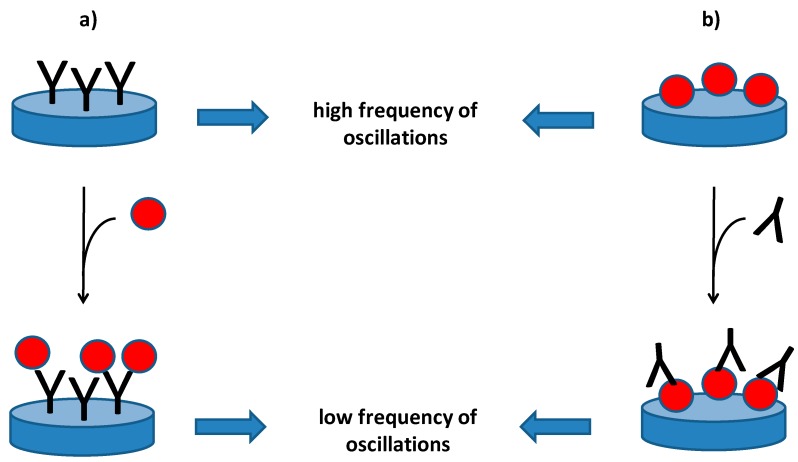
Piezoelectric immunosensors for the determination of an antigen (**a**) or an antibody (**b**). A piezoelectric crystal is depicted as a blue disc. The antibodies are shaped like Y letter because of typical appearance of common immunoglobulins; an antigen is drawn as a red ball.

Piezoelectric immunosensors are analytical devices which can be performed for the determination of various macromolecular compounds and microorganisms. The piezoelectric immunosensors are biosensors which contains an antibody as a biorecognition element and specificity of the antibody significantly influences specificity of the whole immunosensor. In a general expression, a piezoelectric immunosensor is specific when the used antibody is specific and electrode with the other sensitive parts of the piezoelectric material will not be sensitive to unspecific reactions with interfering compounds. Though immunosensors typically contain immobilized antibody and they are able to recognize antigens, the oppose reaction is possible as well. It means that the immunosensor can contain immobilized antigen and be used for the recognition of an antibody and just antibody is the analyzed molecule. The piezoelectric immunosensors with an immobilized antigen are therefore a suitable tool for diagnosis of infectious diseases. Principle of the piezoelectric immunosensors is depicted as Figure 1.

**Figure 2:**
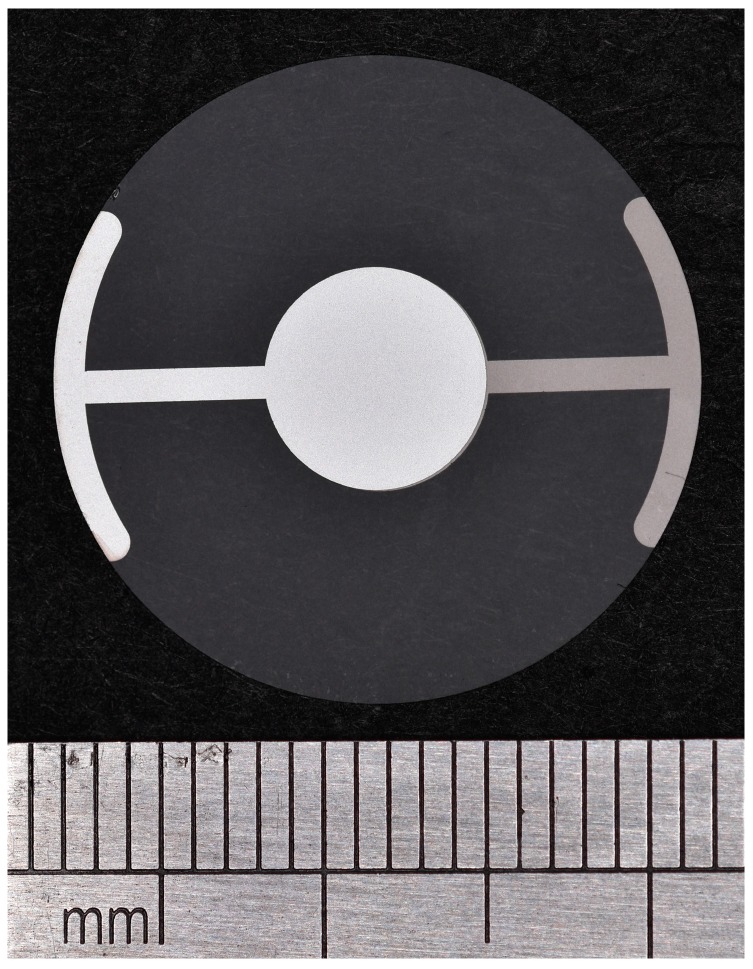
Photograph of a commercially available Quartz Crystal Microbalance sensor with silver electrodes. Scale in millimeters is in the bottom of the photograph.

The piezoelectric immunosensors were researched for a long time period and various promising applications were established as apparent from the following text. Quartz crystal microbalance (QCM) platform is quite popular for the purpose of immunosensor construction which is caused by the fact that QCM are widely used in electronic devices and are commercially available. Example of an QCM is depicted as Figure 2. In the first adaptation discussed here, label free assay of albumin was constructed by Muratsugu and coworkers [18]. The authors decided to prepare an immunosensor for the determination of albumin in urea (albuminuria) and they used a QCM with immobilized antibody against human serum albumin. As a result of the assay, albumin in a range 0.1–100 µg/mL was successfully determined. In another work, complement C4 was determined by a QCM immunosensor where working electrodes were firstly modified with nafion membrane an then by an antibody against complement C4 [19]. The complement C4 was determined in a range 0.08–1.6 µg/mL and relative standard deviation for the assay was around five percent. Good example of a QCM immunosensor was described by Funari and coworkers [20] who immobilized spatially oriented antibodies against gluten on gold electrodes and reached limit of detection 4 ppm for the gluten and sensitivity range was between 7.5 and 15 ppm. 

**Figure 3:**
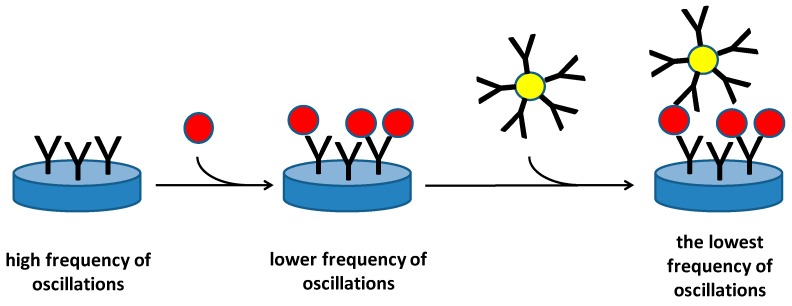
Piezoelectric immunosensors for the determination of an antigen (red ball) and increase of oscillations by application of a nanoparticle (yellow ball) covered with immunoglobulins (Y shaped). Blue disc represents a piezoelectric crystal.

As seen from the cited papers, the piezoelectric immunosensors are suitable for sized analytes with high molecular weight because they cause higher decrease of oscillation frequency. It is also a limitation of such a method, because direct recognition of low molecular weight analytes by antibodies immobilized on a piezoelectric platform is a little complicated. Microorganisms are a specific group of analytes that can be directly analyzed by piezoelectric immunosensors and these analytes are frequently tested by new types of piezoelectric immunosensors [21,22,23]. On the other hand, the assay of microorganisms has its problems too. A microbial cell does not act as an ideal mass point and only the part of the membrane in the proximity of receptor captured by an immobilized antibody is involved in change of oscillations. Though the sensitivity is not ideal for general purposes in health protection, piezoelectric immunosensors can be performed in the protection against biological warfare agents and prevent, for example, military misuse or terrorist activity, by timely detection. Olsen and coworkers prepared an acoustic wave biosensor for *Salmonella typhimurium* by immobilizing of a bacteriophage as a biorecognition element [24]. The biosensor had a limit of detection 100 cells/mL. In another work, immunosensor for the detection of bacteria based on antibodies was constructed on 10 MHz QCMs [25]. The immunosensor contained polyclonal antibody against *Francisella tularensis* immobilized on a self-assembled monolayer and it was able to detect *F. tularensis* with limit of detection 10^5^ colony forming units/mL within 5 min. Further improvement of bacterial assay can be provided by an application of nanoparticles covered by other antibodies against analyte which increases mass on the sensor surface and crosslink the particular bacteria into a firm layer. The principle of such idea is depicted as Figure 3. An assay where analyte was captured within a sandwich between an antibody immobilized on crystal surface and an antibody bound to nanoparticles was successfully tested by some researchers. Salam and coworkers detected *Salmonella typhimurium* by a QCM immunosensor and they used gold nanoparticles covered with antibody for signal improvement [26]. The reached limit of detection in a range of 10–20 colony forming units per ml is very impressive for an immunoassay and the authors were able to reliably analyze the bacteria in food samples. A similar concept was adopted by Guo and coworkers who detected Escherichia coli O157:H7 by a QCM immunosensor using antibody functionalized gold nanoparticles as an amplifier of signal [27]. Limit of detection for the assay started at 10 colony forming units per ml like the previous example. The amplification of signal by modified nanoparticles can be based on the magnetic one because the magnetic nanoparticles contains iron oxides which are known for high mass density. Just the high specific weight is an ideal parameter for a piezoelectric assay. Ferromagnetism of the particles is not necessary and it is not typically involved in the assay thought it can be advantageous, for example, for microfluidic organization of the whole assay. The concept to use magnetic nanoparticles covered with antibodies was described in a study for the detection of tumor necrosis factor α and limit of detection 1.62 pg/mL as well as full correlation to the standard immunological method was reported in the paper [28].

Antibodies are other analytes that can be measured by immunosensors therefore antigen is a typical biorecognition element in the immunosensor. The direct detection of antibodies has been described in some papers and appears to be promising for routine diagnosis of infectious and autoimmune diseases. The diagnosis of infectious diseases based on the determination of antibodies against Human Immunodeficiency Virus [29], antibodies against *Leishmania chagasi* [30], antibodies against *Francisella tularensis* [31], and total immunoglobulins G [32]. 

The described immunosensors are competitive to the standard immunochemical methods. Enzyme-Linked Immunosorbent Assay (ELISA) and Lateral Flow Immunoassay can be designated as the major alternatives for the piezoelectric immunosensors. ELISA is a typical laboratory method and kits for various markers are available in the current market. ELISA is a method readily for a high number of particular analyses with affordable costs. On the other hand, ELISA is based on a number of incubations where samples and secondary labelled antibodies are applied. Application of chromogenic reagents and other supporting chemicals like sulfuric acid is common in the final step where coloration arises. A piezoelectric immunosensor can provide similar analytical parameters like ELISA but there is not a necessity to apply specific reagents or their number is lower comparing to the ELISA because the immunosensors are suitable for a label free assay. Therefore, the piezoelectric immunosensors are able to compete with ELISA. Lateral Flow Immunoassays are other competitors to the piezoelectric immunosensors. Pregnancy test which is an assay of human chorionic gonadotropin in the urine is a typical example of the Lateral Flow Immunoassay. Compared ELISA, the Lateral Flow Immunoassays are significantly simpler and more suitable for use outside laboratories. In total, one step where sample is applied is necessary for the Lateral Flow Immunoassay to be finished. On the other hand, the Lateral Flow Immunoassays are not readily to provide quantification of the analyte concentration. The comparison presented here can be concluded by a statement that the piezoelectric immunosensors can be comparable with ELISA in regards of quality and contemporary to tests like the Lateral Flow Immunoassay in regards of the method simplicity. 

## 3. Molecularly Imprinted Polymers on Piezoelectric Platform

Molecularly Imprinted Polymers are specific artificial materials that can substitute antibodies or antigens as a biorecognition part in a biosensor. The Molecularly Imprinted Polymers are synthesized in the presence of the target molecule (template) which will be analyzed in the next steps. The template can be the same compound as the analyte, but templates with smaller molecular weight than the analyte can be also used when it contains the same specific structural motives. Removal of the template is another step necessary for the Molecularly Imprinted Polymer construction, and success of this step is a condition to achieve functional membrane. Sized templates are hard to be removed from the polymer and physical or chemical processes leading to the removal of the template can also cause destruction of the whole membrane. Only the smaller templates can be removed easier. 

Many materials can serve for the construction of Molecularly Imprinted Polymers, and both organic and inorganic membranes have been proposed for these purposes in the past. Acrylates and acrylamides [33,34], sol-gels [35], chitosan [36,37,38], dextrin [39,40] and organo-metallic composites [41,42] can be exampled as materials with great retaining of the template shape and resistant to the template removing. 

**Figure 4:**
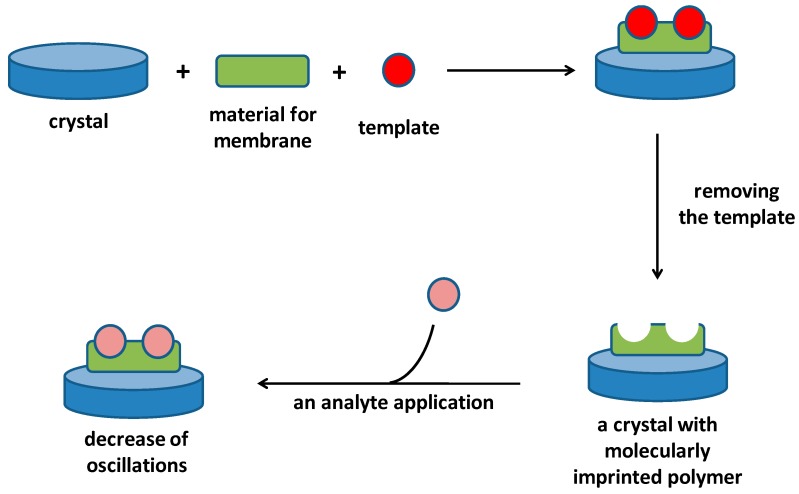
Covering of crystal with a Molecularly Imprinted Polymer and following assay of an analyte chemically identical or close to the template.

The piezoelectric biosensors containing Molecularly Imprinted Polymers are very close to the piezoelectric immunosensors regarding to principle of their use. They directly react with an analyte by an affinity reaction and the affinity reaction causes decrease of the measured oscillation frequency. Application of a specific reagent is not typically necessary in the assay. On the other hand, the assay is also limited by molecular weight of the measured analyte because low molecular weight compounds have lower impact on the oscillation frequency. Schematic depiction of a Molecular Imprinted Polymer fabrication on piezoelectric immunosensor and consequent use of it is depicted in Figure 4.

Adaptations of Molecularly Imprinted Polymers for analytical purposes can be learned from following examples. A QCM sensor electrochemically covered with electropolymerized 3-thiophene acetic acid with an imprint of cytostatic drug melphalan [43]. The electropolymerization was make on the gold electrode of a QCM sensor and the process was controlled by cyclic voltammetry and repeated cycles were admitted. The assay exerted limit of detection 5.40 ng/mL which is promising and it can stand comparison with the common immunoassays. Larger analytes can be also measured by piezoelectric immunosensors. Gupta and coworkers chosen *Neisseria menigitidis*, a meningococcal bacterial causative agent of invasive meningococcal disease, as an analyte [44]. The authors worked with *N. meningitidis* strain MC58 and they chose methacrylic acid, ethylene glycol dimethacrylate and azoisobutyronitrile for the Molecularly Imprinted Polymer which was subsequently created on a QCM sensor. Proteins originated from *N. meningitidis* MC58 which can be discovered in blood serum were fully detected by the sensor. A QCM sensor was electrochemically polymerized with a film of I-methionine with imprinting of taurine [45]. This sensor provided limit of detection 0.12 µmol/L for a taurine solution. Other low molecular weight compounds were successfully analyzed by piezoelectric sensors with Molecularly Imprinted Polymers. The determination of chloramphenicol using polymer based on trimethylopropane trimethacrylate [46] and the determination of caffeine using co-polymerizing methacrylic acid and ethylene glycol dimethacrylate in the presence of azobis(isobutyronitrile) as initiator [47] can be written as examples of sensors based on a 10 MHz QCM sensor. Molecularly Imprinted Polymers can serve also as a recognition tool for specific sequences of genetic information. Bartold and coworkers decided to prepare a tool for the recognizing of single nucleotide polymorphism by the imprinted polymers [48].

## 4. Genetic Information Using Piezoelectric Biosensors 

Genetic information can be employed as a biorecognition part of various biosensors. Single-strand short strains of DNA or RNA can by written down as typical examples of genetic information forms that are suitable for biosensors construction. On the other hand, the whole chromosomes are also good for following of specific interactions [49,50]. Genetic information from a pathogen and human tissue or blood sample are common analytes for these biosensors. However, carcinogens interacting with immobilized chromosomes or double stranded DNA chains can be mentioned as a possible pair: analyte and the biorecognition part of the biosensor [50,51,52,53]. 

**Figure 5:**
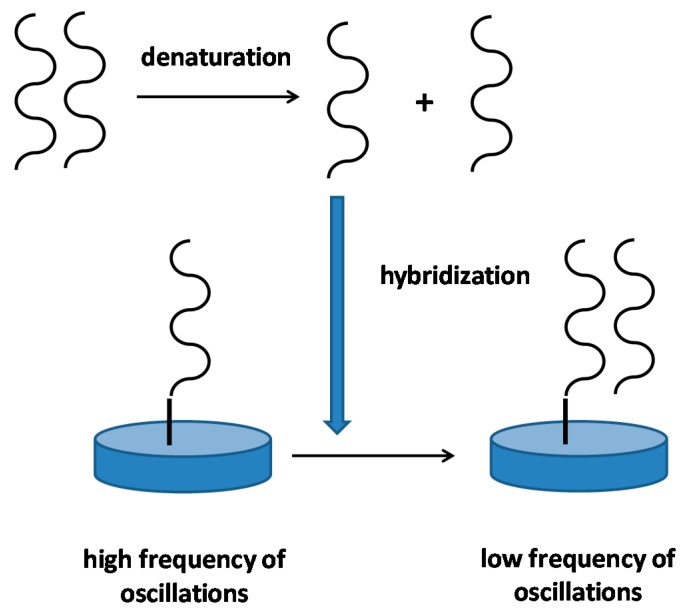
Principle of a DNA piezoelectric biosensor. The picture represents an idealized biosensor, real biosensors can be modified in their principle. In the first step, double stranded DNA in a sample is denatured by, for example, heat. In the second step, single stranded DNA hybridizes with complementary DNA strand immobilized on a piezoelectric sensor. The hybridization results in decrease of oscillation frequency.

The use of RNA and DNA as biorecognition elements has two major practical advantages. Firstly, the chains can be easily prepared by Polymerase Chain Reaction hence fabrication of a biosensor can be done in small companies without work with viable organism or hazardous materials. Secondly, the biosensor can be improved by enlarging the length of the used chain. The second step can take place when false positivity of assay occurs and interference should be suppressed. A simplified principle of a DNA piezoelectric biosensor is depicted as Figure 5.

The piezoelectric biosensors recording hybridization has good properties consisting from a quickly established equilibrium of interaction and capturing of the single stranded chain from solution, high sensitivity and low interference. The study by Kirimli and coworkers can be exampled [54]. The investigators prepared an 8 µm thick lead magnesium niobite-lead titanate piezoelectric plates and immobilized a probe DNA on them. They were able to prove the target DNA with limit of detection 10^−19^ mol/L in urine samples during a half hour period. The piezoelectric immunosensors with embedded genetic information are a good tool for the identification of pathogenic microorganisms [55]. The idea of a DNA-detecting biosensor was adopted in some works. The study of Lian and coworkers can be written down [56]. The authors developed a piezoelectric biosensor containing an aptamer crosslinked by 4-mercaptobenzene-diazonium tetrafluoroborate on graphene interdigitated gold electrode on a crystal and performed it for the detection of *Staphylococcus aureus*. The piezoelectric biosensors revealed presence of *S. aureus* due to the specific capturing of the bacterium on aptamer which caused the DNA bases interaction with *S. aureus* instead of the graphene surface. As low as (limit of detection) 41 CFU/mL was detected by the biosensor. Piezoelectric-excited cantilever sensor with genetic probe for the recognizing of stx2 gene served as a platform for a biosensor detecting *Escherichia coli* O157:H7 [57]. The *E. coli* O157:H7 is one of the Shiga toxin expressing bacteria hence the assay is highly important for control of food that can be contaminated by this bacterial strain. In the described experiment, genomic DNA was isolated from samples containing bacteria and stx2 gene presence was determined. Limit of detection for the method was equal to 700 cells/mL. The authors also made comparison with an immunoassay and constructed a piezoelectric-excited cantilever sensor with immobilized antibodies and collated with the DNA assay with immunoassay. The limit of detection exerted for the immunosensor was 2500 cells/mL which is significantly higher number that for the DNA biosensor. The QCM-based DNA sensors have good premise to became early warning analytical tools for recognizing highly infective microorganisms and viruses during hygienical countermeasures or during military operations, when there is suspicion of an attack by biological warfare agents. The detection of dengue virus can serve as an example [58]. In the cited paper, oligonucleotide functionalized gold nanoparticles helped to amplify signal provided by interaction of dengue virus with surface of QCM. The genome of virus actually served as a bridge for layer by layer deposition of the modified gold nanoparticles. The method exerted promising limit of detection 2 plaque forming units (PFU) per ml and linear correlation in a range 2–2 × 10^6^ PFU/mL. 

**Figure 6:**
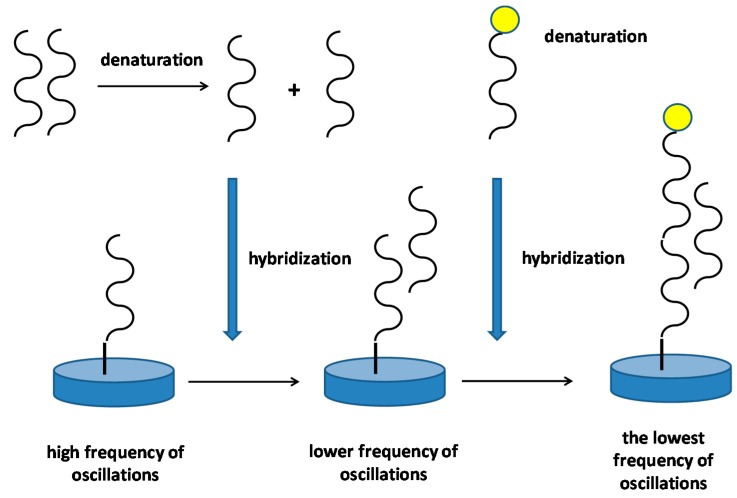
Principle of a DNA piezoelectric biosensor that use a DNA probe labelled with nanoparticles for signal amplifying. In the first step, double stranded DNA in a sample is denatured by, for example, heat. In the second step, single stranded DNA hybridizes with complementary DNA strand immobilized on a piezoelectric sensor. The hybridization results in decrease of oscillation frequency. In the second step, a DNA probe labeled by a nanoparticle is applied and the frequency of oscillations drop again.

The DNA biosensors have good applicability for the diagnosis of genetically determined diseases. Some examples can be introduced as promising studies in this field. Pang and coworkers decided to analyze point mutations in an codon DC17 of the beta-thalassemia gene [59]. The detection was based on hybridization on gold nanoparticles having a DNA probe and DNA in a sample. The hybridization took place on a QCM sensor and the assay had limit of detection 2.6 nmol/L for the tested oligonucleotides. Combination of quantum dots and magnetic nanoparticles was described as a way for the DNA detection in experiment by Ye and coworkers [60]. The authors prepared iron oxide/gold magnetic nanoparticles covered with a DNA probe and CdS quantum dots having single base on their surface. Second probe was a single stranded DNA. The reaction composed from denaturation of a double stranded DNA molecule into single stranded ones. The molecule analyzed reacted with the probe immobilized on iron oxide/gold magnetic nanoparticles. Because the analyzed strain of DNA was longer than the first probe, the remaining chain reacted with the unlabeled second probe. That left a free single base being analyzed for mutations that may finally react with the single base on quantum dots. The whole complex of DNA—magnetic nanoparticles—quantum dots was attracted by a magnet to the surface of a QCM and change in oscillation frequency was measured. The assay had an advantage in that the quantum dots emit strong light when illuminated, and hence both piezoelectricity and fluorescence can be measured. In the aforementioned works, a DNA probe labelled with gold was used as a reagent amplifying the signal (change of oscillations). The principle of the probe function is depicted as Figure 6.

## 5. Other Types of Piezoelectric Biosensors

Apart of the aforementioned common types of piezoelectric biosensors, some alternative proposals deserving consideration were made as well. Lectins are a group of carbohydrate-rich proteins with affinity to sugars. They can act in a similar way to an antibody in immunoassay and they can also exert good specificity to the target structure, and only the carbohydrate moiety in the lectins is crucial for the interactions [61,62]. D-mannose-binding lectin from jackfruit *Artocarpus heterophyllus* served as a recognition part of a biosensor for the determination of *N*-glycosylated receptors on various hematopoietic cells linked to leukemia [63]. In the study, the lectin was anchored to the electrode of a QCM, and it served for the determination of myeloid leukemia cell lines having altered expression of surface glycans. 

Different peptides, cysteinylglycine, glutathione, Cys-Ile-His-Asn-Pro, Cys-Ile-Gln-Pro-Val, Cys-Arg-Gln-Val-Phe, were experimentally tested as the biorecognition parts of biosensors for the detection of volatile compounds in a study by Pizzoni and coworkers [64]. The authors attached the peptides on a 20 MHz QCM and tested deposition of the volatile compounds. They demonstrated readiness of the concept for construction of real gas detector. Volatile chemical compounds including food aromas from classes of alcohols, aldehydes, esters, hydrocarbons and ketones appear to be detectable by this type of biosensor [65,66]. Peptide receptors were also investigated as a platform for the detection of glucose [67]. In the study, short cyclic peptides were immobilized on a QCM and glucose was monitored on the piezoelectric principle on a base of peptide-glucose affinity interaction. 

Piezoelectric biosensors have applicability for the study of biological receptors. Such an array was chosen by Capobianco and coworkers, who decided to analyze epidermal growth factor receptor 2 (Her2) in blood serum samples [68]. The Her2 is a known marker in clinical biochemistry which can help to diagnose certain types of cancer and it has especially good response to breast cancer. In the cited study, a lead zirconate-lead titanate glass piezoelectric microcantilever sensor was prepared and the microcantilevers were covered with antibody against the Her2 and determined on the piezoelectric principle. Concentration of Her2 in a range 0.06–0.6 nmol/L was determined. 

Cofactors and prosthetic groups known from biochemistry can be simply utilized as a recognition parts of a biosensor because of their specificity to enzymatic substrates. Porphyrins are such chemical compounds. In a particular work, electrode of a QCM was covered with tetraphenylporphyrin and adsorption of zinc nitrate was recorded by this piezoelectric sensor [69]. Zinc was captured by the tetraphenylporphyrin and stable complex was formed and easily detectable by the piezoelectric assay. Example papers described in the text above are summarized in Table 1.

## 6. Conclusions

Piezoelectric biosensors are a group of analytical devices suitable for label-free determination of an analyte. The biosensors were experimentally proved reliable and many adaptations are known. Though the commercialization of piezoelectric biosensors has not fully appeared yet, the experimental data are promising and there is expected change in this state in the future. Technologies for mass production of specific materials like nanoparticles, oligonucleotides and imprinted polymers, along with the production of cheap piezoelectric materials, give good presumptions for the next development in this field. Good analytical performance of the described piezoelectric biosensors is of course a condition for the implementation of this technology.

## Figures and Tables

**Table 1:** Survey of papers devoted to biosensors and the other analytical devices using piezoelectric platform.

Recognition Part	Piezoelectric Part	Analyte	Limit of Detection	References
Antibody	QCM ^1^	Albumin in urea (albuminuria)	0.1 µg/mL	[18]
Antibody	QCM	*Francisella tularensis*	10^5^ CFU/mL	[25]
Antibody on the sensor surface and gold nanoparticles covered by antibodies	QCM	Escherichia coli O157:H7	10 CFU/mL	[27]
Molecularly Imprinted Polymer from electropolymerized 3-thiophene acetic acid	QCM	Drug melphalan	5.40 ng/mL	[43]
Electrochemically polymerized I-methionine with molecularly imprinted taurine	QCM	Taurine	0.12 µmol/L	[45]
DNA probe	8 µm thick lead magnesium niobite-lead titanate piezoelectric plates	DNA	10^−19^ mol/L in urine samples	[54]
DNA specific to stx2 gene from *Escherichia coli* O157:H7	Piezoelectric-excited cantilever sensor	*Escherichia coli* O157:H7	700 cells/mL	[57]
Oligonucleotide functionalized gold nanoparticles	QCM	Denque virus	2 PFU/mL	[58]
Antibody	Lead zirconate-lead titanate glass piezoelectric microcantilever sensor	Marker of cancer Her2	0.06 nmol/L	[68]
Various short peptides	QCM	volatile compounds	-	[64]

^1^ QCM—Quartz Crystal Microbalance.
